# Neural Computation of Surface Border Ownership and Relative Surface Depth from Ambiguous Contrast Inputs

**DOI:** 10.3389/fpsyg.2016.01102

**Published:** 2016-07-28

**Authors:** Birgitta Dresp-Langley, Stephen Grossberg

**Affiliations:** ^1^Centre National de la Recherche Scientifique, ICube UMR 7357, University of StrasbourgStrasbourg, France; ^2^Center for Adaptive Systems, Graduate Program in Cognitive and Neural Systems, Department of Mathematics, Boston University, BostonMA, USA

**Keywords:** figure-ground separation, border ownership, perceptual grouping, surface filling-in, V2, V4, FACADE theory, 3D LAMINART model

## Abstract

The segregation of image parts into foreground and background is an important aspect of the neural computation of 3D scene perception. To achieve such segregation, the brain needs information about border ownership; that is, the belongingness of a contour to a specific surface represented in the image. This article presents psychophysical data derived from 3D percepts of figure and ground that were generated by presenting 2D images composed of spatially disjoint shapes that pointed inward or outward relative to the continuous boundaries that they induced along their collinear edges. The shapes in some images had the same contrast (black or white) with respect to the background gray. Other images included opposite contrasts along each induced continuous boundary. Psychophysical results demonstrate conditions under which figure-ground judgment probabilities in response to these ambiguous displays are determined by the orientation of contrasts only, not by their relative contrasts, despite the fact that many border ownership cells in cortical area V2 respond to a preferred relative contrast. Studies are also reviewed in which both polarity-specific and polarity-invariant properties obtain. The FACADE and 3D LAMINART models are used to explain these data.

## Introduction

The non-ambiguous perceptual organization of planar visual images into figure and ground requires the visual system to be able to generate a three-dimensional (3D) representation from a two-dimensional (2D) stimulus input. During viewing of a natural 3D scene, objects that are closer to the viewer may block or occlude the view of objects that are further away. Boundaries of these occluding objects are perceived as belonging to them, a property called *border ownership*. Because occluding objects occur closer in depth than the objects they occlude, border ownership in response to a 3D scene typically coexists with a percept of being closer in depth. The importance of surface border ownership to what may seem nearer to us was already noticed by Galileo (see the review by Dresp-Langley, 2014). The borders of occluding surfaces generally occur in the foreground, while the borders of occluded surfaces generally occur in the background.

**FIGURE 1:**
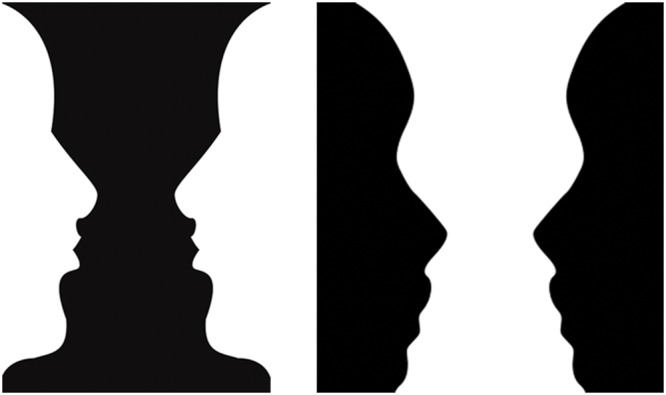
**Two faces or a vase?** In these variations on the famous reversible figures of Rubin (1921), with surface contrasts of opposite signs, the perceptual assignment of border ownership to foreground and background may be influenced by both shifts in spatial attention and prior learning of object categories.

An important problem in visual perception concerns how border ownership assignment occurs in response to 2D pictures, and what role it may play in determining 3D percepts of such pictures. In response to 2D pictures, there are famous examples where the perceptual assignment of surface borders to 3D percepts of foreground and background may be reversible, leading to totally different interpretations of the objects in each representation (**Figure 1**). Such spontaneous changes in figure-ground perception occur only under particular circumstances due to competition between multiple, approximately balanced, 3D interpretations of the 2D image.

During the past half century, many perceptual displays and psychophysical data have described properties of figure-ground perception in response to 2D pictures and 3D scenes. The FACADE (Form-And-Color-And-DEpth) model of 3D vision and figure-ground perception, and its further development and extension by the 3D LAMINART laminar cortical model, have explained and predicted many data about how the brain consciously sees 3D surface percepts in response to 2D pictures and 3D scenes, including, but not restricted to, percepts that involve figure-ground perception (Grossberg, 1994, 1997, 1999, 2014a,b; Grossberg and McLoughlin, 1997; McLoughlin and Grossberg, 1998; Kelly and Grossberg, 2000; Grossberg et al., 2001; Grossberg and Swaminathan, 2004; Yazdanbakhsh and Grossberg, 2004; Cao and Grossberg, 2005, 2012; Grossberg and Yazdanbakhsh, 2005; Grossberg and Hong, 2006; Berzhanskaya et al., 2007; Grossberg et al., 2008; Browning et al., 2009; Fang and Grossberg, 2009; Leveille et al., 2010; Grossberg and Pinna, 2012). Along the way, these models have also explained and predicted many anatomical and neurophysiological data about 3D vision and figure-ground perception in response to both static and moving images and scenes. These explanations involve multiple brain areas, including the lateral geniculate nucleus (LGN) and three parallel cortical streams interacting among cortical areas V1, V2, V4, MT, and MST.

Neurophysiological experiments have also been done to record properties of neurons those activities contribute to figure-ground percepts. In this series, Von der Heydt et al. have published important data in a series of neurophysiological experiments about the border ownership properties of neurons in cortical area V2 of monkeys. In particular, Zhou et al. (2000) reported data from neurons in cortical area V2 that tend to respond to borders with different firing rates depending on whether the border is owned by an occluding or an occluded surface. These neurons are often maximally excited by a preferred combination of direction-of-contrast and border ownership. Zhang and von der Heydt (2010) further studied the contribution of individual edges to border ownership assignment by decomposing figural contours into fragments. Fragments on the preferred side-of-figure produced facilitation, while fragments on the opposite side produced suppression of neural responses. Border-ownership signals also persist for about a second in the brain (O’Herron and von der Heydt, 2009, 2011). Border-ownership signals are generally consistent over multiple variations in shape geometry, configuration, and contrast (von der Heydt et al., 2000; Qiu and von der Heydt, 2005; Qiu et al., 2007). Fang et al. (2009) furthermore used fMRI and found a border ownership BOLD signal in the human visual cortex.

The FACADE and 3D LAMINART anticipated a number of these V2 cell properties, but not all of them. By unifying results from the above-cited theoretical articles with results about how V1 cells that are sensitive to *absolute* binocular disparity are transformed into V2 cells that are sensitivity to *relative* binocular disparity—namely, the difference in absolute disparity of two visible features (Grossberg et al., 2011) — Grossberg (2016) was able to propose a unified explanation of all the main von der Heydt et al. V2 data properties.

As noted above, the von der Heydt et al. data show that various neurons in V2 that are sensitive to border ownership also respond with a preferred contrast polarity. However, the same figure-ground properties can sometimes occur in a given configuration when contrast polarities are mixed, or are switched from one polarity to the opposite, across the stimulus fragments that induce 3D surface percepts (e.g., Mathews and Welch, 1997), and the phenomenal “logic” of such shape percepts (see Pinna and Grossberg, 2006) is indeed likely to involve a complex hierarchy of integration levels in the brain, as explicated by model explanations that involve cortical areas other than V2. The new psychophysical experiments that are reported in this article further probe these intercortical interactions, and illustrate the limitations of explanations that depend exclusively upon V2. Indeed, V2 has been predicted not to directly represent any consciously visible 3D surface qualia, but rather to support amodal object recognition of occluding and partially occluded objects (Grossberg, 1994, 1997, 2014b). The Discussion section explains how and why this may happen as part of a focused summary of the cortical mechanisms that can explain the new data that are reported herein. Before turning to these new results, they are put into a larger historical context with the following partial survey of previous psychophysical and theoretical results.

**FIGURE 2:**
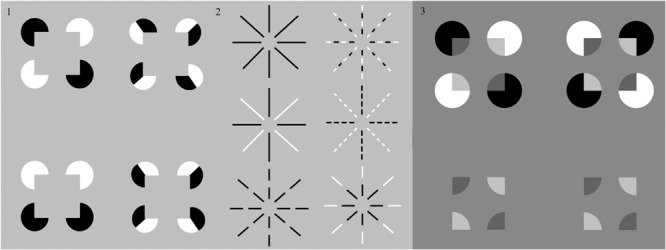
**Four reverse-contrast Kanizsa configurations.** The two Kanizsa squares in the first column represent stimuli used in experiments on sign-invariant boundary detection by Shapley and Gordon (1985) and obey their criteria of sign-invariant boundary induction. The two Kanizsa squares in the second column show cases where single inducing elements are given locally opposing contrast signs, used as stimuli in experiments by Spehar (2000) and Spehar and Clifford (2003). These two configurations do not obey Shapley and Gordon’s criteria of sign-invariant boundary induction. The strength of the illusory boundaries therein was reported to be less discriminable, and even more so when exposure duration was limited to less than 320 ms (Spehar, 2000; Spehar and Clifford, 2003). (2) Six Ehrenstein configurations. The circular illusory surface in the center was reported less perceptible when the radial inducing lines are fragmented, as in the configuration in the right column, and given locally opposing contrast signs, as in the top display of the right column (Dresp et al., 1996; Spehar and Clifford, 2003). When all fragments share the same contrast sign, (as in the bottom display of the left column), the ‘O’ illusion discovered by He and Ooi (1998) is perceived. This percept is abolished when the local contrast signs are of the opposite polarity, [as in the bottom display of the right column. (3) Reverse-contrast Kanisza square inducers can generate a percept of transparency (top row, left column) ir not (top row, right column)]. When the pac men are removed (bottom row), a central rectangular background is perceived to be further away than a surrounding nearer surface with a rectangular aperture. See text for details. [Reprinted with permission from Otsuka et al. (2008)].

The great pioneering work of Kanizsa (1955, 1976,1979, 1985) on subjective contours provided many compelling examples of how illusory surfaces can be induced by spatially sparse, albeit (approximately) colinear, and co-oriented inducers, including examples of figure-ground separation. Prazdny (1983, 1985) additionally noted that the phenomenal strength of surfaces standing out against uniform backgrounds appears as marked in configurations with inducers of opposite contrast polarites as in configurations with inducers of one and the same polarity. Quantitative data for the relative strength of these percepts were not made available in these earlier reports. They were, however, so compelling that they motivated theoretical accounts for boundary detection mechanisms that are insensitive to the local sign of contrast elements in the perceptual assignment of border ownership. Cohen and Grossberg (1984), Grossberg and Mingolla (1985a,b), Shapley and Gordon (1985), and Dresp and Fischer (2001) all noted, in particular, the conceptual importance of a reverse-contrast Kanizsa square (**Figure 2**, part 1) as an example of long-range grouping across opposite contrast polarities in response to polarity-specific inputs from spatially disjoint, oriented detectors. In addition to this boundary-grouping property, the percepts of filled-in surface brightness caused by different inducer configurations had also to be explained.

Both the boundary grouping and surface brightness properties were simulated in a series of neural modeling articles from Grossberg and his colleagues; e.g., Cohen and Grossberg (1984), Grossberg (1984), Grossberg and Mingolla (1985a,b) and Grossberg and Todorovic (1988), at around the same time that classical neurophysiological data about how opposite contrast polarity inputs are pooled at V1 complex cells (Thorell et al., 1984), and about how illusory contour formation occurs in cortical area V2 (von der Heydt et al., 1984), supported model predictions of how bipole grouping cells in V2 can pool inputs from V1 simple, complex, and hypercomplex cells, to form long-range groupings from either like, or opposite, contrast polarity inducers. These explanations, however, were restricted to explaining 2D boundary and surface properties. Neural explanations of 3D properties, including 3D figure-ground separation properties, began with the the FACADE model (Grossberg, 1987, 1994, 1997) as additional neurophysiological and psychophysical studies (e.g., Kapadia et al., 1995; Polat and Norcia, 1996; Dresp and Grossberg, 1997, 1999; Wehrhahn and Dresp, 1998) reported more properties of sign-invariant boundary grouping, sensitive to contrast intensities only, in response to inducers of either polarity [see the recent reviews by Dresp-Langley (2015b) and Spillmann et al. (2015)].

The postulate that boundary grouping by the visual system is insensitive to the contrast polarity of its inducers was subsequently challenged by findings from studies by He and Ooi (1998), Spehar (2000) and Spehar and Clifford (2003), with new configurations where the contrast polarity varies repeatedly within one and the same inducing element. In these cases, the strength of induced perceptual boundaries, or illusory contours, was found to be significantly diminished, especially at stimulus durations shorter than 300 ms (e.g., Spehar and Clifford, 2003). In contrast to examples like the reverse-contrast Kanizsa square, these authors created patterns where the local signs cancel each other out locally, not globally, along an axis of boundary induction (**Figure 2**, part 2). These studies hark back to earlier observations on the Ehrenstein illusion (Dresp et al., 1996), where the perceptual strength of the centrally induced surface does not depend on the contrast polarity of the inducing lines, provided the contrast sign is homogenous within a given inducing element. When the inducers are fragmented into several parts with variable contrast signs (e.g., **Figure 2**, part 2, upper right display), considerably weaker groupings are found. He and Ooi (1998) reported a new ring-shaped illusion, the ‘O’ illusion (**Figure 2**, part 2, lower left display), which is only perceived in fragmented radial lines of one and the same polarity. These findings suggest that the ways in which contrast polarity variations are locally distributed, and the exposure duration of the stimuli, matter critically in the perceptual genesis of shape illusions. At identical physical luminance, opposite contrast signs within one and the same local inducing element may largely cancel each other out and become less effective in perceptual grouping when viewing durations are not long enough. Analogous effects of local contrast changes on long-range perceptual groupings may be observed in percepts of Glass patterns and reverse-contrast Glass patterns (Glass and Perez, 1973; Prazdny, 1984), and can be explained by simular boundary and surface interactions (Cruthirds et al., 1991).

The general theme of different effects of spatially short-range vs. long-range effects of same-polarity vs. opposite-polarity inducers on perceptual grouping and figure-ground perception has a long history, both experimentally and theoretically. Such differences exist in response to both static and moving displays and have led to a large literature about how short-range and long-range filters and grouping mechanisms work together to generate percepts. In the case of static form perception, simple cells in cortical area V1 typically respond to one contrast polarity, but not its opposite, whereas complex cells pool signals from pairs of like-oriented but oppositely polarized simple cells to begin the process of contrast-invariant boundary grouping. Theoretical explanations of these interactions are by now well known in the literature (see Discussion below). Key classical data and neural explanations of how mixtures of contrast-dependent and contrast-invariant mechanisms influence percepts ranging from spatial location and hyperacuity (Badcock and Westheimer, 1985a,b) to brightness perception (Yarbus, 1967; Cornsweet, 1970) can, for example, be found in Grossberg and Mingolla (1985a,b), Grossberg (1987), and Grossberg and Todorovic (1988).

Otsuka et al. (2008) and Spehar and Halim (2016) have presented additional displays in which same-contrast and opposite-contrast inducers can lead to different effects. In particular, the displays in **Figure 5** from Otsuka et al. (2008) are shown in **Figure 2**, part 3. The display in the first row, left panel, generates a percept of unimodal transparency, with an emergent square surface lying in front of four partially occluded pac man figures. The two white pac men are more luminous than the gray background, whereas the two black pac men are less luminous than the background. The illusory Kanizsa square that emerges in this percept thus bridges between opposite-polarity inducers. Opposite polarity inducers also occur in the display in the first row, right panel, but no percept of transparency obtains.

**FIGURE 3:**
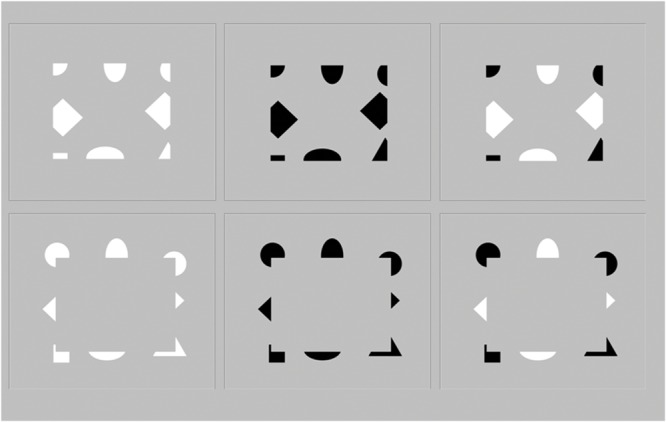
**Six visual configurations presented in the psychophysical experiment.** They generate unambiguous figure-ground percepts of continuous surfaces in depth. In the upper row of these images, the outward-directed contrast edges make the central surface more likely to be seen as lying “behind” the surrounding surface, whereas in the lower row of images, the inward-directed edges make the central surface more likely to be seen as standing out “in front” of” the surround, as explained in the Discussion section by FACADE and 3D LAMINART dynamics confirmed by the experimental data.

Grossberg and Yazdanbakhsh (2005) have explained the percepts that are generated by displays of this kind by simulating how, just by varying the relative contrasts of regions in a display, without changing their geometrical relationships, one can cause a percept of unimodal transparency, bistable transparency, or of a flat surface. **Figure 2** (part 3, top row) illustrates two of these possibilities. A key factor in determining whether such a display looks transparent or not is whether the curved pac man boundary segments at each side of a Kanizsa square boundasry segment have the same contrast, or opposite contrasts, relative to the background. In **Figure 3** (part 3, top row, left), the answer is “same” from pac man to background, and transparency is perceived. In **Figure 2** (part 3, top row, right), the answer is “opposite” and no transparency is perceived.

Many researchers have noted how contrast relations within an image can cause or eliminate a percept of transparency (e.g., Metelli, 1974; Beck et al., 1984; Watanabe and Cavanagh, 1993a,b; Anderson, 1997; Adelson, 2000). Grossberg and Yazdanbakhsh (2005) explain these different percepts using the full machinery of cortical area V1, V2, and V4 interactions within the 3D LAMINART model, with a key role in these transparency vs. no-transparency percepts predicted to be played by a like-polarity competition among simple cells in layer 4 of cortical area V1. Grossberg and Yazdanbakhsh (2005) also summarize neuroanatomical and neurophysiological evidence for all these interactions, but no experiments have yet been done to try to manipulate a transparency percept in animals by altering the strength of this V1 inhibitory interaction.

The displays in **Figure 2** (part 3, bottom row) are derived by removing the outer pac man shapes from the displays in **Figure 2** (part 3, top row). The resulting identical displays generate a percept of an inner background rectangle that is further away than the open rectangular figure that surrounds it. This kind of display is explained in the Discussion section in the same way that the percepts that are generated by **Figure 3** (top row) in the new experiments are explained.

The displays used in the current experiments (**Figure 3**) do not change polarity on a spatial scale within the size of individual simple cell receptive fields. Moreover, these displays are conceptually and mechanistically more challenging to explain than previously tested configurations, and the percepts that they generate are quantified in the *Experimental Results*. The displays used here specifically tested for figure-ground assignment in terms of what is seen as standing out “in front” and what is seen as as “lying behind” by creating configurations in which inducers of varying sign were displayed on either of two sides of a perceptual boundary while the contrast sign within one and the same inducing element was always homogenous. In these configurations, the orientation, direction and polarity of contrast are locally controlled, and may be mixed or switched from one direction and/or polarity to the opposite across the stimulus elements that produce the resulting figure-ground percept. The duration of presentation was not limited in time, as in natural free viewng conditions. An alternative forced choice task similar to that from earlier studies was employed (e.g., Dresp et al., 2002; Dresp-Langley and Reeves, 2012, 2014).

A key variable of the FACADE theory relative to the orientation of surface-inducing contrast edges was tested by presenting inducing elements with outward-oriented contrast edges (**Figure 3**, top row) as well as inducers with inward-oriented edges (**Figure 3**, bottom row). The former case induces percepts with the inducers seen “lying behind” within a closed rectangular background region. This situation requires a subtle analysis of the intercortical mechanisms that are responsible for the depth, surface, and persistence properties of such a background region.

## Materials and Methods

The psychophysical experiments were conducted in accordance with the Declaration of Helsinki (1964) and with the full approval of the corresponding author’s institutional (CNRS) ethics committee. Informed written consent was obtained from each of the participants of the psychophysical experiments. Experimental sessions were run under laboratory conditions of randomized free trial-by-trial image viewing using a Dell PC computer equipped with a mouse device and a high resolution color monitor (EIZO LCD ‘Color Edge CG275W’). This screen has an in-built calibration device which uses the Color Navigator 5.4.5 interface for Windows. The images were generated in Photoshop using selective combinations of Adobe RGB increments to generate contrast inputs (see also Dresp-Langley, 2015a). The luminance levels for each RGB triple could be retrieved from a look-up table after calibration and the values were also cross-checked on the basis of standard photometry using an external photometer and adequate interface software (Cambridge Research Instruments).

### Subjects

Ten unpracticed observers, mostly students in computational engineering who were unaware of the hypotheses of the study, participated in the experiments. All subjects had normal or corrected-to-normal visual acuity.

### Stimuli

The stimuli (**Figure 3**) consisted of six images with different edge contrast inputs. The luminance of the background was 50.5 cd/m^2^ (148,148,148 RGB) in all eight images. The luminance of the black contrast fragments was 1.5 cd/m^2^ (0,0,0 RGB) and the luminance of the white contrast fragments was 99.5 cd/m^2^ (255,255,255 RGB), yielding perfectly balanced Weber contrasts (L_feature_–L_background_/L_background_) of –0.97 and 0.97 for negative and positive polarities in the six images with the fragmented edge contrasts. The height of the central surfaces was 10 cm on the screen, whereas the width was 12 cm. In the six images with the ambiguous fragmented edge contours, about 50% of the inner surface contour was void of a contrast, so that 50% of the boundary contour had to be completed perceptually (Dresp, 1997).

### Task Instructions

A classic psychophysical forced choice procedure with three response alternatives was used to measure perceptual decisions for relative depth (figure-ground). Observers were asked to indicate whether the central surface appeared to “stand in front” of, to “lie behind”, or to be in the “same plane” as the surrounding surface. It was made sure that all observers understood the instructions correctly before an experimental session was initiated.

### Procedure

Subjects were seated at a distance of 1 m from the screen and asked to look at the center of the screen. The experiments were run in a dimmed room (mesopic conditions), with blinds closed on all windows. The six images were presented in random order for about one second each, and each image was presented four times in a session. Inter-stimulation intervals were measured. They typically varied from one to three seconds, depending on the observer, who initiated the next image presentation by striking a key on the computer keyboard. The experiment produced a total of 300 observations from 30 trials per subject in an individual session.

## Results

The individual data from this depth judgment experiment were analyzed in terms of conditional response frequencies, or the frequencies with which the different perceptual responses (“in front”, “behind”, “same plane”) occurred within a given experimental condition. These frequency distributions, permit conclusions relative to event saliency, and allow plotting probabilities (e.g., Overall and Brown, 1957), based on the assumption that a similar frequency distribution is statistically likely to occur in any study population with the same characteristics as the sample population selected for this experiment. To assess whether the observed differences between the response frequencies reflecting the most salient events were statistically predictable, we fed the frequency distributions for “in front” and “behind”, which reflect complementary dimensions of the underlying psychological decision, into analysis of variance (ANOVA) using *Systat 11* (see also Dresp et al., 2002, or Dresp-Langley and Reeves, 2012, 2014). The balanced 2x3 factorial design, with stimuli presented in random order, allowed for generation of psychophysical judgements from an even number of independent forced-choice trials per factor level. Criteria for parametric testing, including normality and egality of variance of the frequency distributions, were met.

### Experimental Results

**FIGURE 4:**
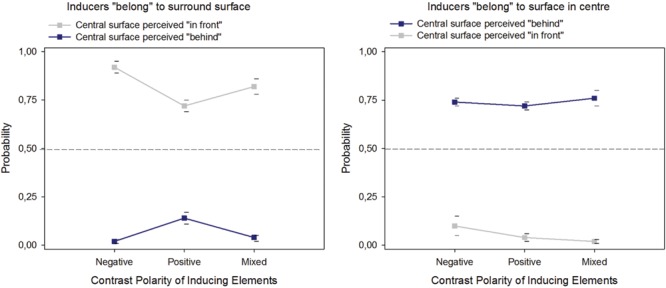
**Probabilities of perceptual decisions for figure (“in front”) or ground (“behind”) assignment of the surface in the center of the images with fragmented edge contours, plotted as a function of the direction of the local edge contrasts and their contrast sign**.

The results (**Figure 4**) show that the configurations generate a higher event probability for the central surface to be perceived as figure (“in front”) when the local contrast edges of the fragmented contour elements are inward directed, as indicated by the distribution of the response frequencies *RF*, with the following average values: *RF*_(infront)_ = 0.83 (SEM = 0.05), *RF*_(behind)_ = 0.07 (SEM = 0.03), and *RF*_(same)_ = 0.10 (SEM = 0.04). The configurations generate a higher event probability for the central surface to be perceived as ground (“behind”) when the local edges are outward directed: [*RF*_(infront)_ = 0.06 (SEM = 0,02), *RF*_(behind)_ = 0.75 (SEM = 0,03), *RF*_(same)_ = 0.19 (SEM = 0,04)]. These perceptual decisions do not depend on the contrast signs of the local edges. Configurations with negative like-contrasts, positive like-contrasts and mixed contrast polarities produced similar response frequency distributions, with average values as follows: *RF*_(infront)_ = 0.51 (SEM = 0,14), *RF*_(behind)_ = 0.48 (SEM = 0,12), and *RF*_(same)_ = 0.10 (SEM = 0,04) for negative like-contrasts; *RF*_(infront)_ = 0.42 (SEM = 0,14), *RF*_(behind)_ = 0.43 (SEM = 0,13), and *RF*_(same)_ = 0.15 (SEM = 0,04) for positive like-contrasts; *RF*_(infront)_ = 0.43 (SEM = 0,11), *RF*_(behind)_ = 0.42 (SEM = 0,10), *RF*_(same)_ = 0.15 (SEM = 0,05) for mixed polarities.

ANOVA on the response frequencies for “in front” and “behind” for the two levels of the factor “contrast edge direction” and the three levels of the factor “contrast sign” returned statistically significant effects of “contrast edge direction” on perceptual decisions for “in front” [*F*(1,2) = 228.30, *p* < 0.001] and “behind” [*F*(1,2) = 212,77, *p* < 0.001]. As expected (e.g., Dresp et al., 2002), no effect of contrast sign on either type of perceptual decision [*F*(1,2) = 2.58, NS on response frequencies for “in front” and *F*(1,2) = 0.25, NS on response frequencies for “behind”] was observed.

## Discussion

**FIGURE 5:**
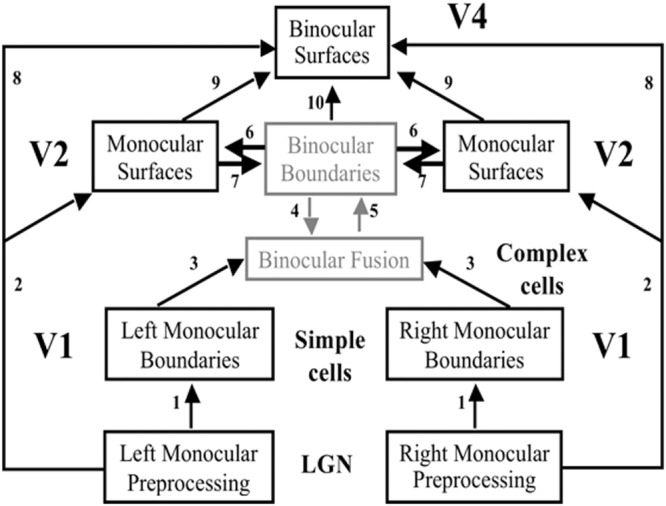
**The FACADE model macrocircuit.** The illuminant-discounted inputs from the Right and Left Monocular Preprocessing stage, which is composed of center-surround cells, output to the Left and Right Monocular boundaries composed of simple cells via pathways 1. Left and Right Monocular Boundaries are binocularly fused via pathways 3. Pathways 4 and 5 complete these boundaries using bipole grouping at the Binocular Boundaries stage. Depthful binocular boundaries mutually interact with the Monocular Surfaces stage (pathways 6), where the closed boundaries are filled-in by the illuminant-discounted surface input. The attached boundaries to the successfully filled-in surfaces generate surface contour output signals. These signals strengthen the boundaries that induced them, and prune the redundant boundaries at the same positions and further depths (pathways 7). The Binocular Surfaces stage binocularly fuses excitatory inputs from the Left and Right Monocular Preprocessing stages (pathways 8) while surface pruning occurs of redundant feature contours at further depths (pathways 9). Boundary enrichment of the Binocular Boundaries occurs at the Binocular Surfaces and regulates surface filling-in there (pathways 10). Boundaries are enriched by adding boundaries at same positions from near depths to far depths. Due to surface pruning, the illuminant-discounted surface inputs that are contained by the enriched boundaries are pruned from the further depths where boundaries are added.

**FIGURE 6:**
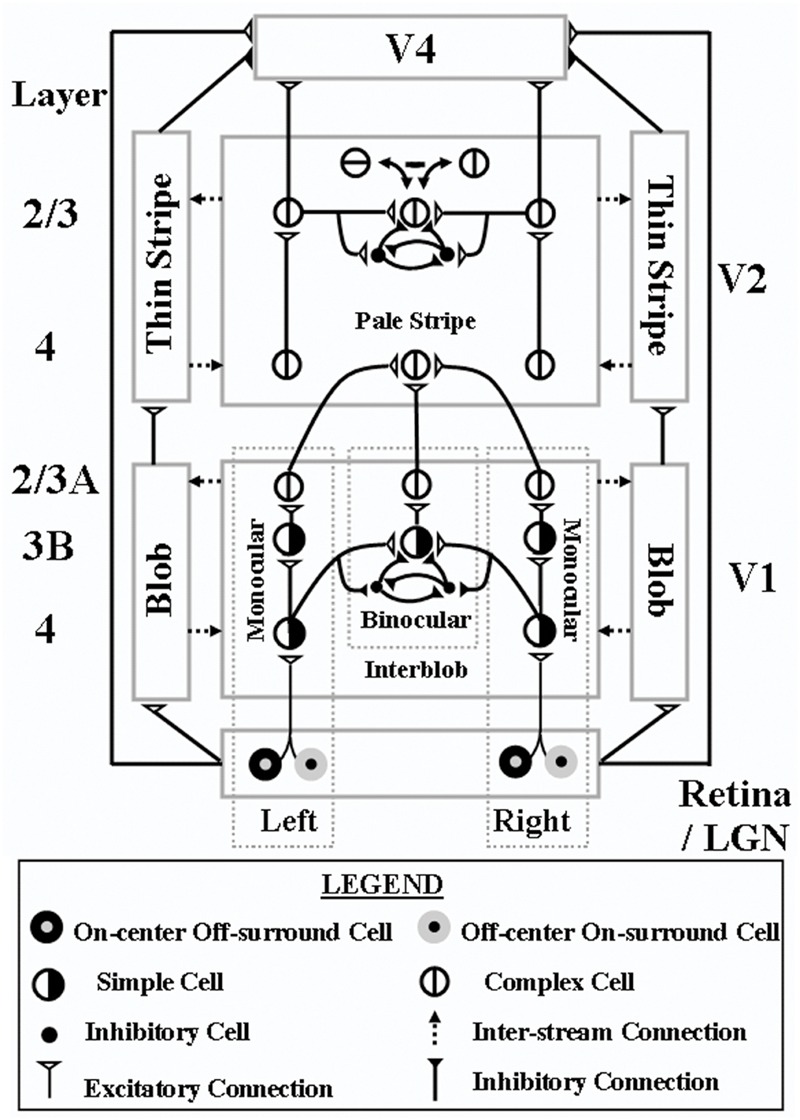
**3D LAMINART model circuit diagram.** This laminar visual cortical model consists of a boundary stream that includes V1 interblobs, V2 pale stripes (also called interstripes), and part of V4, and computes 3D perceptual groupings in different scales; and a surface stream that includes V1 blobs, V2 thin stripes, and part of V4, and computes 3D surfaces that are infused with lightness in depth. Both the boundary and surface streams receive illuminant-discounted signals from LGN cells with center-surround receptive fields, and both converge in V4, where visible 3D surfaces are consciously seen that are separated from their backgrounds. Models V2 and V4 also output to inferotemporal cortex (not shown), where object recognition takes place. Model V1 interblobs contain both monocular and binocular cells. Binocular simple cells become disparity-sensitive by binocularly matching left and right scenic contours with the same contrast polarity in layer 3B before pooling opposite polarity responses at complex cells in layer 2/3A. Monocular and binocular boundary cells control filling-in of monocular 3D surfaces within V1 blobs. Closed boundaries can contain the filling-in process, and can send feedback to V1 interblobs that selectively strengthens the closed boundary components. Monocular and binocular V1 boundaries are pooled in V2. V2 pale stripes can complete 3D perceptual groupings while inhibiting false binocular matches using the disparity filter to solve the correspondence problem. These completed boundaries form compartments in the V2 thin stripes within which filling-in of monocular 3D surfaces occurs. Closed boundaries can contain the filling-in process and send surface-to-boundary surrface contour feedback signals to enhance their generative boundaries, while also suppressing redundant boundaries at the same positions and frrther depths. These conmpleted boundaries and filled-in surfaces complete the representations of partially occluded objects. They do not generate visible percepts, but can be recognized by activating inferotemporal cortex. Visible surfaces in which figures are separated in depth from their backgrounds are formed in V4. Here, left and right eye feature contour signals from the LGN are binocularly matched, while redundant feature contour signals are pruned at further depths by inhibitory signals from the thin stripes. Then the pruned feature contour signals induce filling-in of a visible surface percept within enriched binocular boundaries. V4 emits output signals that lead to recognition and grasping of unoccluded parts of opaque surfaces – Reproduced with permission from Fang and Grossberg (2009).

A unified mechanistic explanation is here provide of the percepts induced by the **Figure 3** images using FACADE and 3D LAMINART model mechanisms (**Figures 5** and **6**). In particular, model mechanisms are summarized with enough detail to achieve a self-contained explanation of the new data using boundary and surface stream interactions within and between cortical areas V1, V2, and V4, while also clarifying the insufficiency of V2 neurophysiological data about border ownership to explain the resulting conscious percepts.

### Bipole Boundary Completion Can Pool Over Opposite Contrast Polarities

**FIGURE 7:**
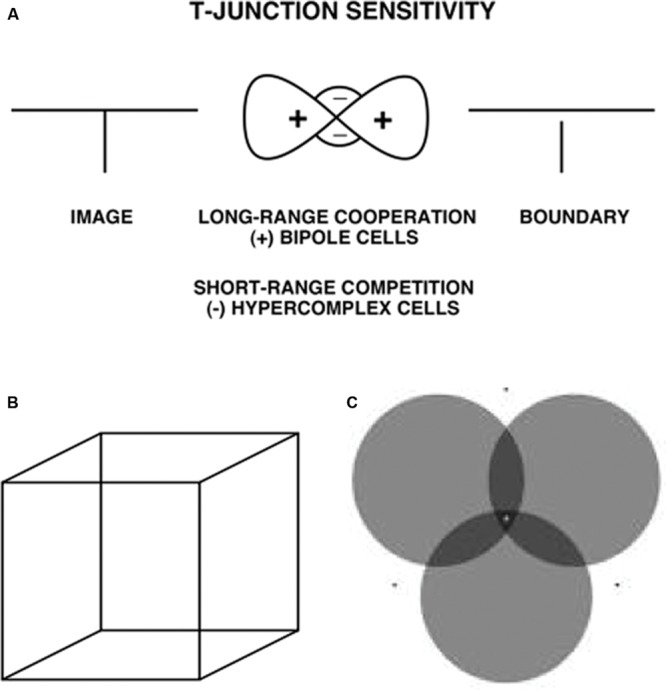
**(A)** T-Junction Sensitivity. (left) T-junction in an image; (middle). Bipole cells provide long-range cooperation (+), and work together with inhibitory interneurons that provide cells provide short-range competition (-); (right). An end gap in the vertical boundary arises because, for cells near where the top and stem of the T come together, the top of the T activates bipole cells along the top of the T more than bipole cells are activated along the T stem. As a result the stem boundary gets inhibited whereas the top boundary does not – Reprinted with permission from Grossberg (1997) – **(B)** Necker cube. This 2D picture can be perceived as either of two 3D parallelograms whose shapes flip bistably through time. **(C)** When attention switches from one circle to another, that circle pops forward as a figure and its brightness changes. See Grossberg and Yazdanbakhsh (2005) for an explanation – Reprinted with permission from Tse (2005).

In response to all of the images in **Figure 3**, boundaries can be completed inwardly between pairs of adjacent colinear inducers. The completion process uses the oriented long-range horizontal cooperation of *bipole grouping* cells in layer 2/3 of cortical area V2, balanced by shorter-range disynaptic inhibition (**Figures 6** and **7A**). Bipole cells can complete boundaries in response to colinear inducers with the same relative contrasts with respect to the background, as in the leftmost two columns of **Figure 3**, as well as between inducers with opposite relative contrasts with respect to the background, as shown repeatedly in psychophysical experiments (e.g., Wehrhahn and Dresp, 1998; Tzvetanov and Dresp, 2002). This is true because bipole cells receive their inputs, after several stages of additional processing, from complex cells in layer 2/3 of cortical area V1 (**Figures 5** and **6**). Complex cells, in turn, pool inputs from simple cells in layer 4 of V1 that have the same preferences for position and orientation, but opposite contrast polarities. As a result, bipole cells can complete boundaries around objects that lie in front of textured backgrounds whose relative contrasts reverse along the perimeter of the object. In the present cases, bipole cells complete rectangular boundaries that abut all their inducers.

### Bipoles Are Sensitive to T-junctions

The long-range cooperation and short-range competition processes whereby bipoles complete boundaries are sensitive to any T-junctions that lie along the boundaries that they complete (**Figure 7A**). In the images with incomplete boundaries, there are no explicit T-junctions in the image. However, when a rectangular boundary is completed, T-junctions are created at the corners of the colinear inducing contrasts. The bipole cells that lie along the orientation of a completed boundary (the “head” of the T) get more excitatory input than do the bipole cells that lie near the head of the T, but whose orientational preference is along the perpendicular or oblique orientation of the inducing contrast (the “stem” of the T). This is true because the bipole cells that are activated along the head of the T receive strong excitatory inputs from both sides of their receptive fields, whereas the bipole cells that are activated along the stem of the T receive excitatory inputs from just one side of their receptive fields (**Figure 7A**). The more strongly activated bipole cells inhibit surrounding bipole cells more than conversely through a spatially short-range competitive network. As a result, the bipole cells near the head that are along the stem get inhibited. An *end gap* hereby forms in each boundary near where the stem of a T touches its head (**Figure 7A**).

Because the bipole cells can complete rectangular boundaries in response to spatially disjoint inducers with the same relative contrasts with respect to their surrounding regions, or in response to combinations of inducers with opposite relative contrasts, end gaps at the T-junctions can form in either case.

As originally explained in Grossberg (1994, 1997), and simulated in such articles as Kelly and Grossberg (2000), Grossberg and Swaminathan (2004), and Grossberg and Yazdanbakhsh (2005), end gaps trigger a process of figure-ground perception and border ownership in which the rectangular boundaries are often perceived in front of the regions that they enclose, which are themselves perceived as a ground at a slightly further depth. For example, the percepts of the Necker cube (**Figure 7B**) can be explained in this way (see Grossberg and Swaminathan (2004)), as can the way that shifts in attention can make an attended disk in **Figure 7C** look both nearer and darker (Grossberg and Yazdanbakhsh, 2005; Tse, 2005). These concepts are reviewed and extended below in order to explain the conscious 3D surface percepts that are generated by the images in **Figure 3**, notably why the percepts of the completed rectangular surfaces in response to the **Figure 3** (bottom row) displays appear in front of their surrounding regions, but the percepts of the completed rectangular surfaces in response to the **Figure 3** (top row) displays look further away than their surrounding regions.

In order to motivate these theoretical explanations, it is useful to ask the following question: If it is indeed the case that these figure-ground relationships do not depend on having inducers with the same contrast polarity, then why do so many cortical area V2 cells that are sensitive to border ownership also exhibit a particular contrast preference; e.g., Zhou et al. (2000). This can be understood by going into more detail about how end gaps trigger figure-ground perception and border ownership.

### Feedback between Boundaries and Surfaces Achieves Complementary Consistency

The FACADE and 3D LAMINART models (**Figures 5** and **6**) detail how the figure-ground perception process utilizes feedback between the boundary completion process in the interblob cortical stream and the surface filling-in process in the blob cortical stream within V1, V2, and V4 of visual cortex, This feedback enables boundaries and surfaces to generate a consistent percept, despite the fact that they obey computationally complementary laws. This property is called *complementary consistency*. As will be noted shortly, the mechanisms that ensure complementary consistency also contribute to 3D figure-ground separation. Grossberg (2016) explains in detail how the data of von der Heydt et al. about border ownership and related properties of V2 cells fit into this larger theory.

**FIGURE 8:**
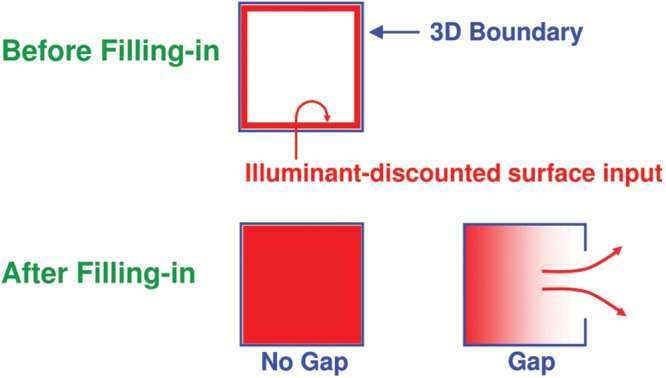
**The top row illustrates how, at a prescribed depth, a closed boundary contour abuts an illuminant-discounted feature contour.** When this happens, the feature contours can fill-in within the closed boundary. The bottom row (left) depicts how filling-in of the feature contours is contained by this closed boundary contour, thereby generating large contrasts in filled-in activity at positions along the boundary contour. Contrast-sensitive surface contour output signals can then be generated in response to these large contrasts. The bottom row (right) depicts a boundary contour that has a big hole in it at a different depth. Feature contours can spread through such a hole until the filled-in activities on both sides of the boundary equalize, thereby preventing contrast-sensitive surface contour output signals from forming at such boundary positions – Reprinted with permission from Grossberg (2016).

In particular, the completed boundaries with their end gaps are projected topographically from the interstripes, or pale stripes, of V2, at which boundaries are completed, to the thin stripes of V2, at which one stage of surface filling-in occurs. When surface filling-in occurs within these boundary inducers, brightness and color can flow out of the end gaps, thereby equalizing the filled-in brightness and color on both sides of the remaining boundaries near these gaps (**Figure 8**, bottom row). Only if the boundary of the rectangle is closed, with no significant gaps, can it fully contain its surface-filling in. In the percepts that are generated by the displays in **Figure 3**, the inducers that are inside or outside these rectangles are surrounded by closed boundaries, since the frame of the image provides another closed boundary that can contain filling-in between it and the bipole-generated rectangular boundary that lies within it. The significance of this fact will be discussed below.

### Closed Boundaries, Surface Contours, and Boundary Pruning

**FIGURE 9:**
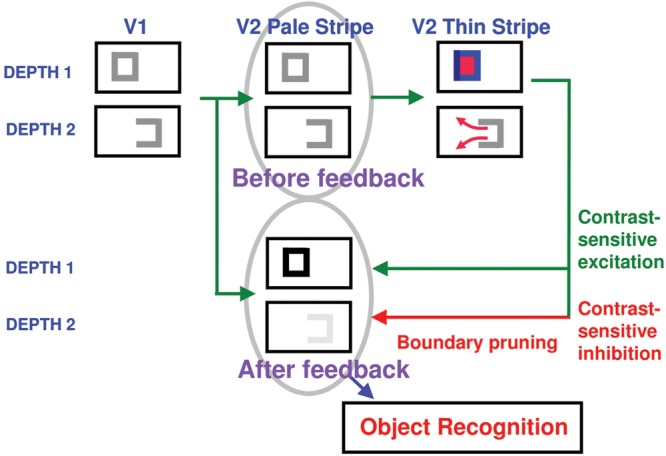
**A closed boundary can form at Depth 1 by combining a binocular vertical boundary at the left side of the square with three monocular boundaries that are projected along the line of sight to all depths.** Surface contour output signals can thus be generated by the FIDO at Depth 1, but not the FIDO at Depth 2. The Depth 1 surface contours excite, and thereby strengthen, the boundaries at Depth 1 that controlled filling-in at Depth 1. These surface contours also inhibit the redundant boundaries at Depth 2 at the same positions. As a result, the pruned boundaries across all depths, after the surface contour feedback acts, can project to object recognition networks in inferotemporal cortex to facilitate amodal recognition, without being contaminated by spurious boundaries – Reprinted with permission from Grossberg (2016).

As filling-in occurs, feedback can occur from the surfaces in the thin stripes to the boundaries in the pale stripes (**Figure 9**). These feedback signals occur from each active Filling-In DOmain, or FIDO. They are *surface contours* that are generated by contrast-sensitive on-center off-surround networks that act across position and within the depth represented by each FIDO. These contrast-sensitive networks sense sufficiently large and steep spatial discontinuities in the filled-in brightnesses or colors within their FIDO. They hereby generate surface contour output signals only at the surface positions that are surrounded by closed boundaries. In response to the incomplete inducers in the top row of **Figure 3**, these regions lie on both sides of the completed boundaries. However, due to the end gaps, surface contour signals are not generated at the boundary positions of the inducers themselves.

The surface contour output signals generate topographic feedback signals to a subset of the boundary representations that induced them (**Figure 9**). These feedback signals are delivered to the boundary representations via an on-center off-surround network whose inhibitory off-surround signals act within position and across depth (**Figure 9**). The on-center signals strengthen the boundaries that generated the successfully filled-in surfaces at the same depth, whereas the off-surround signals inhibit spurious boundaries at the same positions but further depths. This inhibitory process is called *boundary pruning*. Surface contour signals hereby strengthen consistent boundaries and prune, or inhibit, redundant boundaries.

Because surface contour signals are generated by the contrasts of a filled-in surface, they are sensitive to a particular contrast, but not to the opposite one. Their feedback to boundaries thus makes the responses of the recipient bipole cells also sensitive to this contrast, even though the bipole cells, in the absence of surface contour feedback signals, respond to both contrast polarities, due to their inputs from V1 complex cells, so that they can complete boundaries of objects in front of textured backgrounds. Thus, both surface contour signals and their target bipole cells also exhibit sensitivity to a particular contrast polarity, as in the neural data of Zhou et al. (2000).

In response to 3D scenes, boundary pruning is part of the process of *surface capture* whereby feature contours can selectively fill-in visible surface qualia at depths where binocular fusion of object boundaries can successfully occur. Boundary pruning helps to strengthen closed boundaries, while competitively eliminating boundaries with gaps, leaving the closed boundaries to contain the filling-in process and to thereby support depth-selective surface percepts. Surface contour and boundary pruning signals hereby work together to generate 3D percepts based on successfully filled-in surface regions.

For example, the open boundary at Depth 2 in V1 and the V2 pale stripes of **Figure 9** can be created due to a monocularly viewed vertical boundary that is seen by only one eye, as occurs during daVinci stereopsis (Nakayama and Shimojo, 1990; Gillam et al., 1999; Cao and Grossberg, 2005), and by a pair of horizontal boundaries that do not give rise to strong binocular disparities. Such depth-non-selective boundaries are projected to all depth planes along the line of sight (Grossberg and Howe, 2003; Cao and Grossberg, 2005). The closed boundary at Depth 1 in **Figure 9** is due to these boundaries plus a left vertical boundary that is formed at that depth due to binocular disparity matching between the two eyes. As a result of surface filling-in Depth 1 of the V2 thin stripes, and the resultant formation of surface contours only at Depth 1, the closed boundary at Depth 1 is strengthened, whereas the spurious open boundary at Depth 2 is inhibited by the on-center off-surround surface contour feedback signals within position and across depth from V2 thin stripe surfaces to V2 pale stripe boundaries.

### From Boundary Pruning to Figure-Ground Separation

**FIGURE 10:**
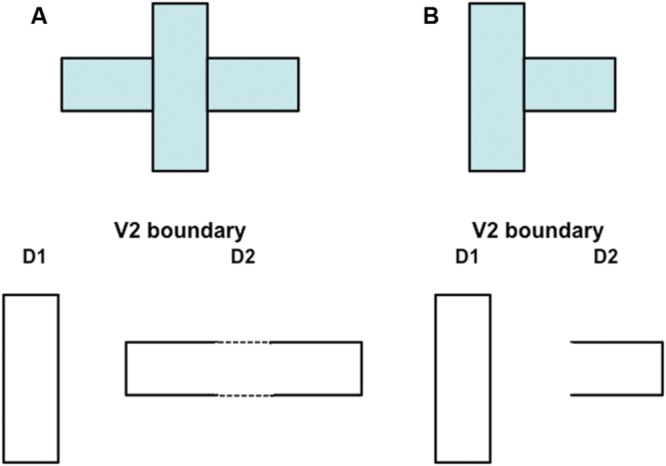
**Initial steps in generating a 3D percept of figures at different depths in response to a 2D picture with particular occlusion. (A)** This figure is composed of three abutting rectangles but generates a percept of a vertical rectangle that partially occludes a horizontal rectangle. Due to mechanisms described in the text, the boundary of the vertical rectangle is separated onto a near depth D1 and achieves border ownership of its shared boundaries with the two smaller rectangles. The remaining boundaries are separated onto a slightly further depth D2, where they can use bipole completion to complete the boundary of the partially occluded horizontal rectangle (dotted lines). This picture does not show the boundary fragments at depth D1 in which end gaps have been generated. The text and **Figure 11** 10 propose how end gap boundaries are eliminated. **(B)** This figure is composed of two abutting rectangles. Although there is no completion of the horizontal rectangle behind the vertical rectangle, a 3D percept can nonetheless be generated using mechanisms summarized in **Figure 11** and the surrounding text.

Remarkably, by eliminating the spurious boundaries, the off-surround signals that are activated by surface contours also enable figure-ground separation to proceed. They do so by separating occluding and partially occluded surfaces onto different depth planes, after which partially occluded boundaries and surfaces can be amodally completed behind their occluders without interference from the now-inhibited spurious boundaries. For example, the three rectangles in **Figure 10A** are perceived as a vertical rectangle in front of a partially occluded horizontal rectangle. Due to the action of surface contours, the redundant copy of the vertical rectangle at a further depth (denoted by D2 in **Figure 10A**) is inhibited, thereby enabling the horizontal boundaries corresponding to the smaller rectangles to be colinearly completed within depth D2. In response to the picture in **Figure 10B**, the redundant vertical rectangular boundary is inhibited at depth D2, thereby restoring the boundary fragments at depth D2 that previously were inhibited by the D2 vertical boundaries at end gaps. For this reason, end gaps are not seen in the final depthful percept.

The above interactions help to explain how, in response to the images in **Figure 3**, the inducers always appear to lie on a surface behind an occluding surface. Whether the end gaps form inside an illusory rectangle, as in response to the images in **Figure 3** (top row) or outside an illusory rectangle, as in response to the images in **Figure 3** (bottom row), they will be seen as further away than the surface that contains no end gap boundaries.

Further analysis is, however, needed to explain how any surfaces are consciously seen—since, as further explained below, V2 boundaries and surfaces are predicted to support recognition, but not conscious seeing, of completed occluders and their partially occluded objects—and also to explain how spurious end cut boundary fragments at both depths do not interfere with the recognition process.

### How the Disparity Filter Eliminates Some Spurious Boundaries in the Near Depth

Although the boundaries containing end-gaps in response to the displays in **Figure 10A** are eliminated by surface contours at the further depth D2, they are not eliminated in this way from depth D1. These near-depth boundary fragments are eliminated by the disparity filter (**Figure 6**), an inhibitory circuit in layer 2/3 of V2 that operates along the line of sight and across depth to help solve the correspondence problem (Grossberg and McLoughlin, 1997; Grossberg and Howe, 2003; Cao and Grossberg, 2005). In particular, the D1 near-depth end gap horizontal boundaries are inhibited by the D2 far-depth rectangular boundaries in **Figure 10** at corresponding positions by the disparity filter. This happens because the D2 far-depth rectangular boundary can be completed after surface contour signals act from the D1 closed vertical rectangular boundary to inhibit the spurious D2 vertical boundaries at the same positions. The completed D2 far-depth horizontal rectangular boundary can then contain an amodal surface filling-in process, and can generate its own surface contour signals. In contrast, the D1 end gap horizontal boundaries remain, and no boundary strengthening occurs along them. As a result, the D2 horizontal rectangle boundaries can inhibit the D1 end gap horizontal boundaries via the disparity filter, more than conversely.

Although the disparity filter can eliminate the near-depth end gap horizontal boundaries in response to the image in **Figure 10A**, it cannot do so in response to the image in **Figure 10B**. This is because the D2 far-depth boundary is not closed in this case after surface contour signals act from the D1 vertical rectangular boundary, and thus is not strengthened by its own surface contour feedback signals. The same kind of situation occurs in response to the fragmented inducers in **Figure 3**. How, then, are end gap near-depth D1 horizontal boundaries eliminated in this case?

### From Unoccluded and Occluded Recognition in V2 to Unoccluded Seeing in V4

In order to explain how these spurious boundaries are also eliminated, it needs to be explained how additional mechanisms generate the *modal*, or consciously visible, percepts of the unoccluded parts of both occluding and occluded objects in depth. FACADE theory proposes how boundaries and surfaces may be *amodally* completed in V2 for purposes of recognition, but also that conscious qualia of the unoccluded surfaces of opaque objects are predicted to be represented in V4 due to a *surface-shroud resonance* that is triggered between V4 and the posterior parietal cortex (PPC); see Grossberg (2013) for a discussion of these resonant dynamics and the data that they help to explain. These proposed V2 and V4 representations enable the brain to complete the representations of partially occluded objects behind their occluders in V2 for purposes of object recognition, without forcing all occluders to appear transparent, which would be the case if the completed boundaries and surfaces that are illustrated in **Figure 10A** could generate visible surface qualia. How these V2 and V4 mechanisms may cooperate to achieve both effective recognition and seeing were first described in Grossberg (1994, 1997) and then further developed and simulated in many further articles; e.g., Kelly and Grossberg (2000) and Fang and Grossberg (2009). As noted above, Grossberg and Yazdanbakhsh (2005) additionally explained and simulated how both opaque and transparent percepts can be generated using the same model cortical dynamics.

Before summarizing these V2-to-V4 mechanisms for conscious seeing, it is worth noting here that surface contour signals also help to control where the eyes look and to thereby help to regulate how the brain learns invariant object categories. The first role arises because surface contour signals are strongest at the distinctive features of an attended object, such as at high curvature positions along a boundary. In addition to the (thin stripe)-to-(pale stripe) feedback that enhances some boundaries while pruning others, a parallel pathway, that is predicted to occur through cortical area V3A, clarifies how these enhanced surface contour positions can also determine target positions of eye movements that explore an attended object’s surface. These signals are proposed to determine where the eyes will look next on an attended surface, and thereby enable inferotemporal cortex to learn view-, size-, and positionally-invariant object categories as the eye movements explore this surface. Thus, the 3D LAMINART model is part of a more comprehensive 3D ARTSCAN Search architecture for active vision wherein 3D boundary and surface representations help to control eye movements for attending, seeing, searching, learning, and recognizing invariant object categories (Fazl et al., 2009; Grossberg, 2009; Cao et al., 2011; Foley et al., 2012; Chang et al., 2014; Grossberg et al., 2014).

### Boundary Enrichment and Surface Pruning in V4

To set the stage for explaining these V2-to-V4 processes, keep in mind that the boundary pruning process spares the closest surface representation that successfully fills-in at a given set of positions, while removing redundant copies of the boundaries of occluding objects that would otherwise form at further depths. This process illustrates “the asymmetry between near and far”. When the competition from redundant occluding boundaries is removed, the boundaries of partially occluded objects can be amodally completed behind them on boundary copies that represent further depths, as in the percept induced by the display in **Figure 10A**. Moreover, when the redundant occluding boundaries collapse, the redundant surfaces that they momentarily supported collapse as well. Occluding surfaces are hereby seen to lie in front of occluded surfaces.

These surface representations in V2 are depth-selective due to their depth-selective capture by binocular boundaries, but they do not combine brightness and color signals from both eyes (**Figure 5**). They are said to be computed within *monocular* Filling-In-DOmains, or FIDOs. The computation of binocular surfaces that combine brightness and color signals from both eyes is proposed to take place in V4 (**Figure 5**). These networks are called *binocular* FIDOs. Here monocular surface signals from both eyes are binocularly matched (pathways 8 in **Figure 5**). The successfully matched binocular signals are pruned by inhibitory signals from the monocular FIDOs (pathways 9 in **Figure 5**). These sur*face pruning* inhibitory signals eliminate redundant feature contour signals at at their own positions and further depths. As a result, occluding objects cannot redundantly fill-in surface representations at multiple depths. This surface pruning process is a second example of the “the asymmetry between near and far”.

As in the case of the monocular FIDOs, the feature contour signals to the binocular FIDOs can initiate filling-in only where they are spatially coincident and orientationally aligned with binocular boundaries. Boundary pathways 10 in **Figures 5** and **6** hereby carry out depth-selective surface capture of the binocularly matched feature contour signals that survive surface pruning. In all, the binocular FIDOs fill-in feature contour signals that: (a) survive within-depth binocular feature contour matching (via pathways 8) and across-depth feature contour inhibition (via pathways 9); (b) are spatially coincident and orientationally aligned with the binocular boundaries (pathways 10); and (c) are surrounded by a connected boundary, or fine web of such boundaries.

**FIGURE 11:**
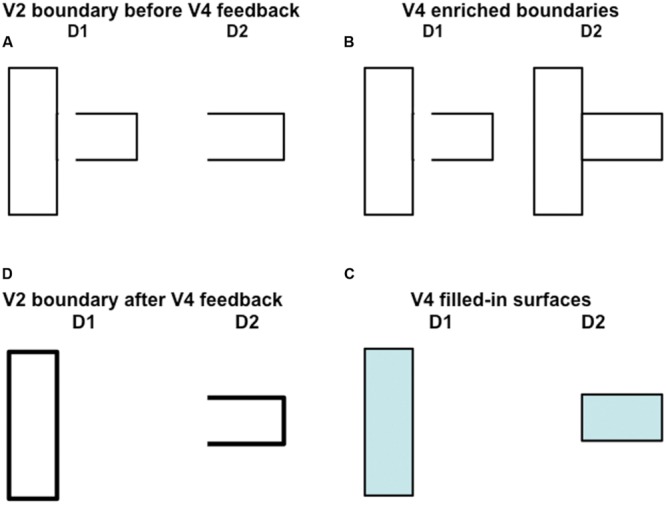
**How spurious end gap boundaries are eliminated.** This figure illustrates how spurious end gap boundaries are eliminated from the near depth D1 in the 3D percept that is generated by the 2D picture in **Figure 10B**. In this case, the end gap boundaries at depth D1 in **(A)** cannot be eliminated, as they can in response to the percept generated by **Figure 10A**, by the disparity filter in V2 after surface contour feedback strengthens closed boundaries at the pale stripes from thin stripes. This is true because the boundary at depth D2 is not closed; see **(A)**. On the other hand, this boundary is closed by boundary enrichment in V4; see **(B)**. As a result, top-down attention from the filled-in surfaces in V4 (see **(C)**) can strengthen the boundaries of closed regions in V2 (see thicker lines in **D**). After this happens, the disparity filter in V2 can eliminate the end gap boundary at depth D1 in **(A)**.

In addition, at the binocular FIDOs, the binocular boundaries of nearer depths are added topographically to those that represent further depths (e.g., **Figure 11B**). This third instance of the asymmetry between near and far is called *boundary enrichment*. When the vertical right boundary of the vertical rectangle at depth D1 in V4 enriches the boundaries at depth D2, a closed horizontal rectangular boundary is completed at D2, as shown in **Figure 11C**. This closed boundary can then modally fill-in its surface brightness at D2. These enriched boundaries prevent opaque occluding objects, such as the D1 vertical rectangle in **Figure 11C**, from looking transparent by duplicating its boundaries at further depths, and thereby blocking filling-in of occluded objects behind them, much as the horizontal rectangle at D2 is prevented from filling-in behind the vertical rectangle at D1 in **Figure 11C**.

The total filled-in surface representation across all binocular FIDOs—after all three processes of boundary pruning, surface pruning, and boundary enrichment act—represents the visible surface percept. It is called a FACADE representation because it combines properties of Form-And-Color-And-DEpth. As to the three asymmetries between near and far, it is possible that they arise during development due to the asymmetric optic flows that are caused by moving forward much more than backward.

### Top–Down Attention from V4 to V2 Eliminates End Gap Boundaries via Disparity Filter

As noted above, although the disparity filter can eliminate the D1 near-depth end gap horizontal boundaries in response to the image in **Figure 10A**, it cannot do so in response to the image in **Figure 10B** because the D2 far-depth boundary is not closed in this case after surface contour signals act from the D1 vertical rectangular boundary, and thus is not strengthened by its own surface contour feedback signals. The same kind of situation occurs in response to the fragmented inducers in **Figure 3**. Given the above discussion about how V4 boundaries and surfaces form, it is now possible to explain how end gap near-depth D1 horizontal boundaries are eliminated in this case.

This is accomplished by top–down feedback from the V4 filled-in surfaces to their generative V2 boundaries (not shown in **Figure 6**). These top–down signals are contour-sensitive and obey the ART Matching Rule (e.g., Carpenter and Grossberg, 1987, 1991), which predicts how top-down object attention works. The ART Matching Rule is defined by a modulatory on-center, off-surround network. The modulatory on-center can select and gain-amplify features within it, while the off-surround can inhibit features at other positions in the broad off-surround. The predicted properties of this network have been supported by many psychological and neurobiological data, and there is a convergence among models of attention about the mathematical form that the rule should take. See Grossberg (2013) for a review.

In the present instance, the modulatory on-centers of the completed rectangles at each depth, D1 and D2, in V4 can strengthen the corresponding boundaries at their respective positions and depths in V2, while inhibiting other boundaries in their off-surrounds, as illustrated in **Figure 11D**. The disparity filter can then eliminate the spurious end gap boundaries at depth D1 in V2 that are generated by the image in **Figure 10B**.

The 3D boundary and surface representations that are depicted in **Figures 10** and **11** provide an explanation of how the fragmented images in **Figure 3**, each of whose inducers is caricatured by the image in **Figure 10B**, generate their depthful figure-ground percepts, notably why the relative depths of figure and ground depend on the positions of the T-junctions relative to the completed boundaries, but not on the relative inducer contrasts that caused them. In response to the fragmented images in **Figure 3**, these boundaries need to be completed by bipole grouping cells before T-junctions can be created at the fragmented inducers, unlike in response to the images in **Figure 10**. Once that happens, surface filling-in within closed boundaries ensues. **Figures 10** and **11** clarify how the boundary and surface representations within V2 can lead to recognition of figure and ground objects in V2, without these representations also leading to visible surface qualia (see **Figure 9**). The filled-in surface representations within V4 are predicted to support conscious percepts of the qualia of the unoccluded parts of opaque surfaces, while their boundaries also enhance the strength of the boundary fragments at corresponding positions in V2.

Although the present exposition focuses on the perception of opaque surfaces in V4, both unique and bistable transparent percepts have also been explained by these FACADE and 3D LAMINART mechanisms (Grossberg and Yazdanbakhsh, 2005).

## Conclusion

This article presents additional experimental evidence to complement the fact that many cells in cortical area V2 that are sensitive to border ownership, and thus implicated in the process of figure-ground perception, also exhibit a preferred contrast polarity. The experimental results here, with configurations that match previously established criteria for sign-invariant boundary grouping, show that contrast polarity is often unimportant in determining what part of a 2D picture generates a 3D percept of a closer figure, and what part generates a 3D percept of a further background. Both same-polarity and mixed-polarity sets of figural inducers, with either darker or lighter contrasts compared to the background, can generate the same percepts of relative depth.

The results support the hypothesis that V2 is just one stage in a cortical hierarchy that also includes V1 and V4 in the generation of surface percepts with figure-ground properties. Using model interactions among all of these cortical areas, FACADE theory and the 3D LAMINART model explain the psychophysical experimental data here, as well as many other psychophysical data about 3D vision and figure-ground perception in previously published articles. These models also explain many data about identified cells and circuits in these cortical areas, notably, in Grossberg (2016) all the key V2 data that have been reported in neurophysiological experiments about border ownership and related figure-ground properties by the von der Heydt laboratory.

## Author Contributions

All authors listed, have made substantial, direct and intellectual contribution to the work, and approved it for publication.

## Conflict of Interest Statement

The authors declare that the research was conducted in the absence of any commercial or financial relationships that could be construed as a potential conflict of interest.
